# Remineralization Effect of a Strontium-Containing Composite: An In Vitro Study

**DOI:** 10.3390/ma19091709

**Published:** 2026-04-23

**Authors:** Adriana Martínez-Llop, Jose Luis Sanz, María Melo, Sofia Folguera, Gonzalo Llambés, James Ghilotti

**Affiliations:** Department of Stomatology, Faculty of Medicine and Dentistry, Universitat de València, 46010 Valencia, Spain

**Keywords:** bioactive composites, dentin remineralization, carbonated hydroxyapatite, Fourier transform infrared spectroscopy, energy dispersive spectroscopy

## Abstract

The aim of this in vitro study was to evaluate the ability of the new strontium-containing composite, Stela (SDI, Victoria, Australia), to induce hydroxyapatite formation and promote remineralization of demineralized dentin, compared to SDR Flow+ (York, PA, USA). Twenty-four dentin slices (1 mm thick) were obtained from extracted wisdom teeth using a microtome and demineralized with 17% EDTA for 2 h. A layer of either Stela or SDR Flow+ was applied to each slice, allowed to set, and preserved in 0.1% thymol solution. Samples were analyzed at 1, 7, 14 and 28 days (*n* = 3 per group and time). Measurements were taken at baseline, after demineralization, and after application. Apatite formation was assessed using Fourier-transform infrared spectroscopy (FTIR), while changes in the Calcium/Phosphate (Ca/P) ratio were evaluated by Energy Dispersive Spectroscopy (EDX). Statistical comparisons were performed using the Wilcoxon test (*p* < 0.05). Both materials promoted carbonated hydroxyapatite formation and increases in calcium and phosphate. Stela exhibited an apatite peak (1420 cm^−1^) as early as 24 h and significant increases in calcium and phosphate from day 7. SDR Flow+ reached its peak at 14 days and showed significant increases in the Ca/P ratio. By 28 days, both materials achieved comparable remineralization, confirming their effectiveness in treating demineralized dentine.

## 1. Introduction

Dentin constitutes most of the tooth structure and is composed of approximately 70% mineral material, 20% organic matrix, and 10% water. It is a mineralized tissue, neither vascularized nor innervated, based on collagen, where inorganic apatite crystals are embedded in an extracellular matrix [1]. The loss of its integrity is mainly associated with the appearance of caries: a multifactorial, non-communicable and dynamic disease, mediated by biofilms and modulated by diet. Its development is due to a continuous cycle of demineralization and remineralization of hard dental tissues [2,3]. In the early stages, acids produced by bacterial metabolization of fermentable carbohydrates dissolve calcium and phosphate crystals on the subsurface of the enamel [4]. As the process progresses, dentin collagen is exposed and, in a low-pH environment, matrix metalloproteinases (MMPs) are activated to promote its degradation [2,5].

Under physiological conditions, the processes of demineralization and remineralization remain in balance. Demineralization involves the loss of calcium, phosphate, and fluoride ions along with the dilution of apatite, while remineralization involves their recovery and the crystallization of new apatite. When this balance is disrupted, initial carious lesions develop in enamel [6]. Remineralization is therefore a natural repair mechanism based on the deposition of calcium phosphate, which restores the properties of mineralized tissues [2,7].

In recent decades, restorative dentistry has evolved toward a more conservative approach, following the philosophy of “minimal intervention”. This prioritizes the preservation of healthy tissue and encourages the use of bioactive materials capable of inducing favorable responses in dental tissues [2]. In this context, glass ionomer cements (GICs) have established themselves as fluoride-releasing materials [8,9], and bioactive glass has even been shown to form apatite-like layers on enamel and dentin, promoting remineralization [2,10].

Strontium, an alkaline earth metal with chemical properties similar to calcium, has gained increasing attention in restorative dentistry due to its potential role in dentin remineralization. Strontium ions (Sr^2+^) can partially substitute calcium in the apatite structure, enhancing mineral stability and influencing crystal formation. Additionally, strontium has been associated with biological effects, such as the inhibition of matrix metalloproteinases (MMPs) involved in dentin degradation, and it may act synergistically with fluoride to reduce bacterial activity [8,11,12].

As a result, strontium has been incorporated into various oral care products and biomaterials, including bioactive glasses, hydroxyapatite-based systems, and resin-based composites. Although most evidence comes from in vitro studies, these materials have shown promising results in improving mineral content, reducing lesion depth, and enhancing dentin remineralization. Therefore, strontium is currently considered a bioactive co-remineralizing agent with potential applications in restorative materials [2,13,14].

Among these strontium-containing materials, SDR (Dentsply Sirona, York, PA, USA) and its flowable variant SDR Flow+ (Denstply Sirona, York, PA, USA) have been widely used in restorative dentistry. These materials are based on a modified UDMA resin matrix designed to reduce polymerization shrinkage and incorporate strontium-containing fillers, which may contribute to radiopacity and potential remineralizing effects [15]. Most recently, Stela (SDI, Victoria, Australia) has been introduced as a restorative material that also incorporates strontium within its formulation. According to the manufacturer, it features an optimized particle system aimed at improving mechanical and aesthetic properties. However, independent scientific evidence regarding its demineralizing performance and clinical behavior remains limited.

The development of dental materials that combine functional and bioactive properties is essential to improve clinical outcomes and enhance the long-term preservation of dental tissues. In this context, recently introduced materials incorporating strontium have attracted attention due to their potential remineralizing effects. However, despite these theoretical advantages, the available independent evidence regarding their bioactivity remains limited.

Within this framework, the aim of this in vitro study was to evaluate the potential bioactivity of a strontium-containing restorative material (Stela). Specifically, hydroxyapatite formation on demineralized dentin was assessed using Fourier-transform infrared spectroscopy (FTIR), and changes in elemental composition associated with remineralization were analyzed by energy-dispersive X-ray spectroscopy (EDX). The results were compared with those obtained from another strontium-containing composite (SDR Flow+).

The hypotheses guiding this study are as follows: first, that Stela is capable of remineralizing demineralized dentin (H1), in contrast to the null hypothesis, which states that it is not (H0); and second, that there are significant differences in the dynamics of remineralization and hydroxyapatite formation between Stela and SDR Flow+ at different action times (H1), as opposed to the null hypothesis, which assumes no such differences exist (H0).

## 2. Materials and Methods

The study protocol was approved by the Ethics Committee of the University of Valencia (ID: 2030322). Samples were obtained from impacted third molars extracted for surgical reasons unrelated to caries or periodontal disease. The teeth, free from oral contamination, were stored in 0.1% thymol solution to preserve their structural and chemical properties until the start of the study.

### 2.1. Sample Preparation

For sample preparation, the wisdom teeth were fixed to cyanoacrylate with screws and then carefully embedded in molds with transparent epoxy resin (HeyBalon^®^, Spain), allowing them to set for 24 h. The teeth were sectioned longitudinally with the Mintron Stuers^®^ precision cutting machine (Struers, Ballerup, Denmark) to obtain 1 mm thick sections that included enamel and dentin, which were again stored in 0.1% thymol solution until use. A total of 24 valid samples were obtained.

An initial distribution of the samples was carried out by randomly assigning them to two groups according to the material to be applied: SDR Flow+ (control group; CG) and Stela (experimental group; EG). Each group was then randomly subdivided into four categories according to the material’s action time: 28, 14, 7 and 1 day.

Prior to any treatment, baseline measurements were taken using FTIR spectroscopy and field emission scanning electron microscopy (FESEM) equipped with EDX, obtaining reference data for each sample. Data from the spectroscopy and microscopy equipment is described in Section 2.2 and Section 2.3.

Then, the tooth sections were immersed in 17% Ethylene Diamine Tetraacetic Acid (EDTA) solution (CanalPro^®^, Coltène, Altstätten, Switzerland) for 2 h. After this step, samples were washed and stored again in 0.1% thymol solution, and a post-demineralization measurement was made using the previously mentioned FTIR and FESEM-EDX equipment.

The restorative materials were then applied according to the assigned group, following a uniform protocol (described below) and complying with the manufacturer’s instructions. The composites evaluated in this study, including their composition and respective manufacturers, are summarized in Table 1.

- Stela (EG): The Stela primer was applied on the surface of the dentin sample, waiting 5 s before drying with the air syringe. According to the manufacturer, the Stela Primer is applied to the dentin surface prior to composite placement to enhance adhesion, promote chemical bonding with the tooth structure, and improve the integrity of the dentin–composite interface. Next, the Stela capsule was vibrated for 10 s, and it was placed in a single increment. A waiting time of 15 s was allowed to ensure complete setting.
- SDR Flow+ (CG): A single-step universal adhesive (Prime & Bond Universal; Scotchbond^TM^, 3M, Seefeld, Germany) was applied on the surface of the dentin sample, dried, and light-cured for 10 s. Next, a unidose capsule of SDR Flow+ (Dentsply Sirona^®^, Charlotte, NC, USA) was placed on the sample and light-cured for 20 s using a 1000–1200 mW/cm^2^ LED lamp.

**Figure 1:**
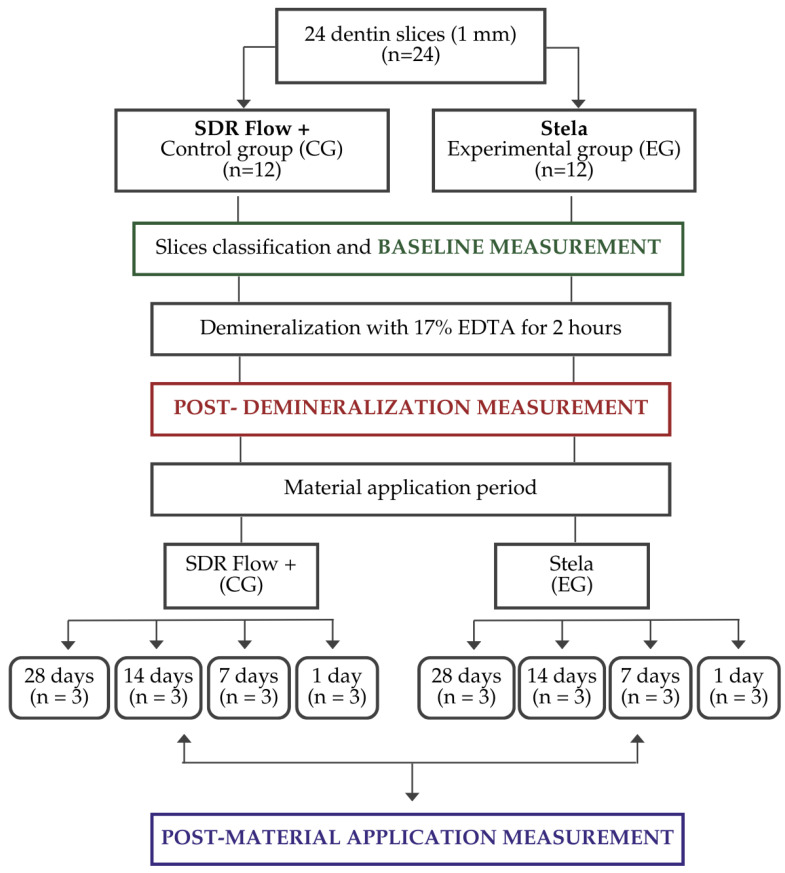
Experimental design of the 24 sections of 1 mm, divided into two groups (Stela and SDR Flow+). Baseline and post-demineralization measurements were performed using FTIR and EDX analyses. Materials were applied according to four subgroups (*n* = 3 per subgroup) based on application time (28, 14, 7 and 1 day), and final measurements were recorded.

The procedure was carried out uniformly in all groups, varying only the action time of the material according to the assigned category (28, 14, 7 or 1 day). After the composite application, the samples were immersed in a Phosphate-Buffered Solution (PBS, pH 7.4) to simulate a physiologically neutral aqueous environment during the observation period. PBS was selected as a standardized and widely used storage medium in in vitro remineralization studies due to its stable ionic composition and neutral pH, which allow ion release from the materials without introducing additional remineralizing agents that could confound the results [16]. Figure 1 provides a graphical representation of the process.

### 2.2. Fourier Transform Infrared Spectroscopy (FTIR)

For this study, a Fourier Transform Infrared Spectrometer (FTIR) equipped with an Attenuated Total Reflectance (ATR) system was used, specifically the Cary 630 FTIR model (Agilent Technologies^®^, Santa Clara, CA, USA). For each analysis, a 1 mm section was placed on the appropriate support, applying uniform mechanical pressure to ensure consistent contact between the sample and the ATR crystal, thereby minimizing variability associated with sample-crystal contact. The infrared laser beam was properly directed onto the thoroughly dried sample, and sample drying was performed systematically to reduce the potential influence of surface moisture on band intensities. A single measurement per sample was performed, which, while consistent with the methodology reported in comparable in vitro remineralization studies [17], represents a limitation of the present study as acknowledged in the limitations section. All measurements were taken from the same surface of each section, specifically the side opposite to that on which the composite was applied, ensuring a standardized measurement area across all samples and minimizing the potential influence of surface roughness variability on spectral intensity. Between samples, the surface of the device was cleaned with 2-propanol to prevent cross-contamination. This technique enabled the evaluation of the light absorbance of the samples. Spectra were recorded in the range of 650–4000 cm^−1^, with a total of 64 scans per measurement at a resolution of 4 cm^−1^. The intervals of the interest were as follows [18]:

- 3000–3700 cm^−1^: OH^−^ group (hydroxyl), direct indicator of hydroxyapatite formation.
- 1636 cm^−1^: COO^−^ group (carboxylate), associated with organic interactions in the remineralization process.
- 1021 cm^−1^: PO_4_^3−^ group (phosphate), key marker of the mineralization and deposition of remineralizing material.
- 820 and 1420 cm^−1^: CO_3_^2−^ group (carbonate), related to carbonated hydroxyapatite.
- 700 cm^−1^: P_2_O_7_ group (pyrophosphate), involved in crystallization control and potential marker of chemical residues.

### 2.3. Energy Dispersive Spectroscopy (EDX)

The SCIOS 2 FIBSEM field emission scanning electron microscope (Thermo Scientific^®^, Waltham, MA, USA) was used, equipped with EDX (X-ray Energy Dispersive Spectroscopy) Ultim Max 170 (Oxford Instruments^®^, Oxfordshire, UK), which allowed us to analyze the elemental chemical composition, identifying and quantifying the elements present in each of the samples.

For each sample, the EDX analysis area was systematically selected on the dentin surface opposite to the composite application site, ensuring a standardized and consistent measurement zone across all samples and time points. This approach was adopted to minimize inter-sample variability associated with area selection and to focus the elemental analysis on the dentin surface most likely to reflect ion diffusion from the overlying material. Specific SEM scanning areas were selected, and these regions were subjected to spot analysis to generate detailed EDX spectra. The spectra obtained represented the intensities of X-rays emitted as a function of energy, which made it possible to identify the characteristic elements present in each sample and calculate their percentage by weight (%wt). In this study, the analysis focused on calcium (Ca) and phosphorus (P), from which the Ca/P ratio was calculated.

### 2.4. Statistical Analysis

This study analyzed the Ca/P ratio for each material in its baseline state and after treatment, considering the application times (1, 7, 14 and 28 days). The normality of the data was evaluated using the Shapiro–Wilk test, but due to the limited sample size, nonparametric tests were used.

The Wilcoxon test was used to compare calcium and phosphate values and their ratio within each material before and after treatment, as well as between the Stela and SDR Flow+ groups, evaluating remineralizing efficacy. A significance level of *p* < 0.05 was considered in all analyses.

## 3. Results

### 3.1. Fourier Transform Infrared Spectroscopy (FTIR)

FTIR results are presented in graphs comparing basal, post-demineralization and post-treatment measurements to evaluate the remineralizing effect of Stela and SDR Flow+. The *Y*-axis represents absorbance and the *X*-axis represents wavenumber (λ; cm^−1^), using color codes: green for basal, red for demineralized and blue for post-treatment. The peaks of the OH, COO^−^, CO_3_^2−^ and PO_4_^3−^ groups of interest in this study are indicated.

**Figure 2:**
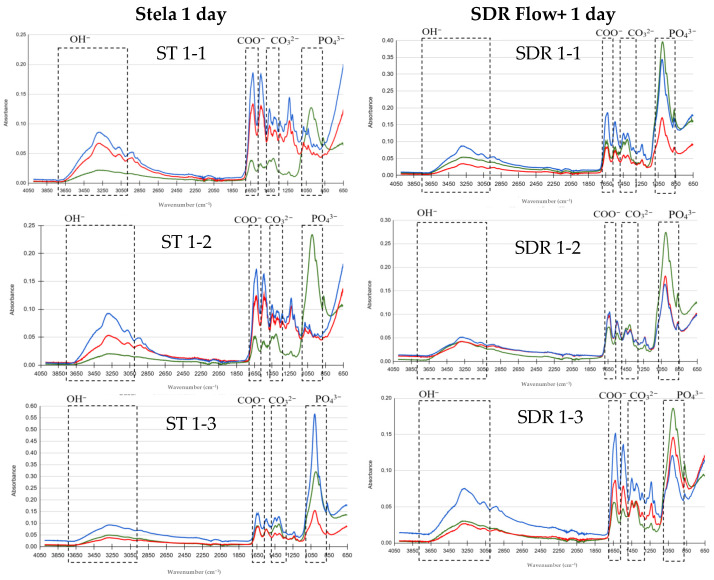
Comparison of FTIR spectra for the Stela and SDR Flow+ groups at baseline, post-demineralization and post-treatment after 1 day. Three samples per group: Stela (ST 1-1, ST 1-2, ST 1-3) and SDR Flow + (SDR 1-1, SDR 1-2, SDR 1-3).

After one day of treatment (Figure 2), FTIR spectra showed a decrease in absorbance after demineralization in both groups. In the samples treated with Stela, the OH^−^ band (3000–3700 cm^−1^) showed similar values between the basal and demineralized phases, with an increase after treatment. Phosphate peaks (~1030 cm^−1^) decreased after demineralization and showed partial recovery, exceeding the baseline level in some samples. The carbonate (~1450 and 1420 cm^−1^) and carboxylate (COO^−^) (~1630 cm^−1^) bands increased after treatment compared to the demineralized state. In the group treated with SDR Flow+, the OH^−^ band showed mild recovery after treatment. Phosphate peaks showed limited recovery, whereas carbonate and carboxylate peaks increased moderately compared to the demineralized phase.

**Figure 3:**
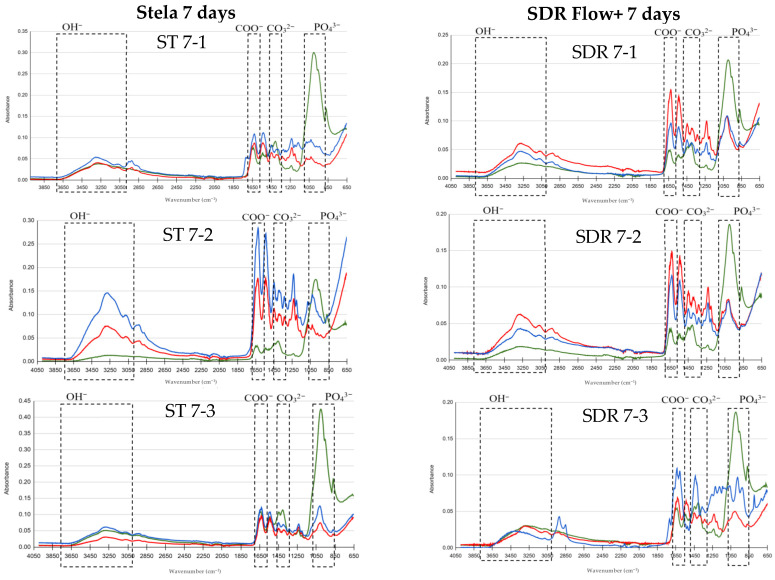
Comparison of FTIR spectra for the Stela and SDR Flow+ groups at baseline, post-demineralization and post-treatment after 7 days. Three samples per group: Stela (ST 7-1, ST 7-2, ST 7-3) and SDR Flow + (SDR 7-1, SDR 7-2, SDR 7-3).

Figure 3 shows FTIR spectra after 7 days of treatment. In the group treated with Stela, the OH^−^ band decreased after demineralization and showed a slight recovery following material application, with similar intensities observed after one day. Phosphate peaks (~1030 cm^−1^) showed partial recovery, approaching the basal level in only one sample (ST 7-2). Carbonate and carboxylate bands exhibited uniform recovery, exceeding basal values in some cases. In the samples treated with SDR Flow+, the OH^−^ band remained similar after demineralization, with slight increases in some samples following treatment. Phosphate peaks increased compared to the demineralized state but did not reach basal values. Carbonate and carboxylate bands showed homogeneous recovery, exceeding initial levels in most samples.

**Figure 4:**
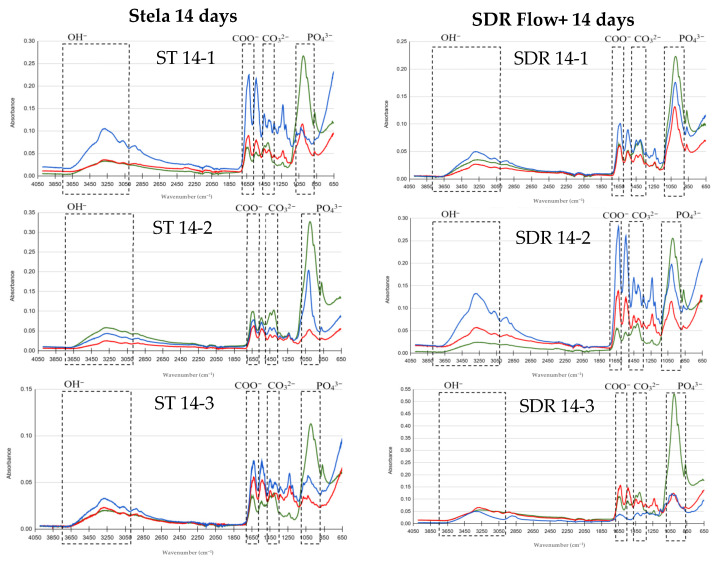
Comparison of FTIR spectra for the Stela and SDR Flow+ groups at baseline, post-demineralization and post-treatment after 14 days. Three samples per group: Stela (ST 14-1, ST 14-2, ST 14-3) and SDR Flow + (SDR 14-1, SDR 14-2, SDR 14-3).

Figure 4 shows the graphs regarding 14 days of treatment. In the samples treated with Stela the OH^−^ band reached values similar to or higher than the basal level in most samples. Phosphate peaks decreased after demineralization and showed progressive recovery following treatment, approaching or exceeding the basal level in some samples (ST 14-2). Carbonate and carboxylate bands exceeded initial values, with absorbance levels higher than those observed at 1 and 7 days. In the group treated with SDR Flow+, the OH^−^ band also reached or exceeded basal values after treatment. Phosphate peaks showed variable recovery, exceeding the basal level in some samples (SDR 14-1, SDR 14-2). Carbonate and carboxylate bands exhibited widespread recovery, surpassing initial values in most samples.

**Figure 5:**
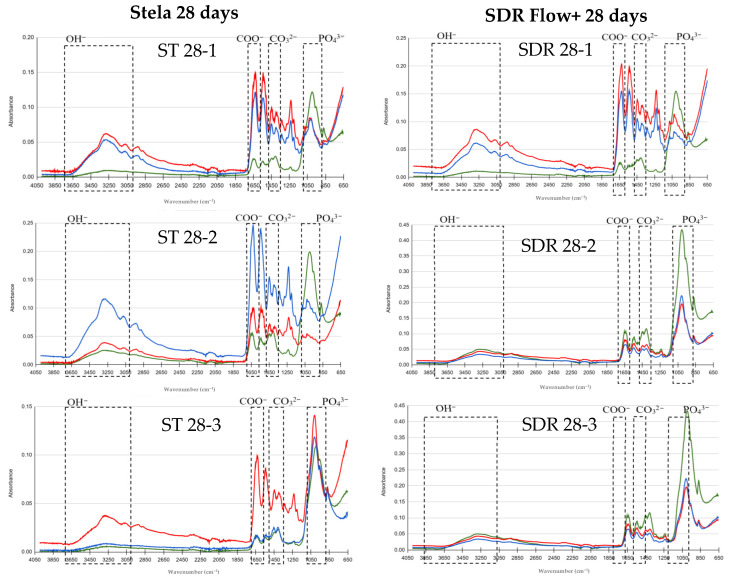
Comparison of FTIR spectra for the Stela and SDR Flow+ groups at baseline, post-demineralization and post-treatment after 28 days. Three samples per group: Stela (ST 28-1, ST 28-2, ST 28-3) and SDR Flow + (SDR 28-1, SDR 28-2, SDR 28-3).

Finally, after 28 days of treatment with Stela, the OH^−^ band showed an increase in absorbance compared to the basal level in all samples. Phosphate peaks inhibited significant recovery following demineralization, reaching values above the basal level in some cases (ST 28-3). Carbonate and carboxylate bands exceeded initial levels, with absorbance values higher than those observed after 14 days of treatment. In the group treated with SDR Flow+, the OH^−^ band showed a general increase in absorbance compared to the basal level. Phosphate peaks exhibited a progressive recovery without reaching initial values. Carbonate and carboxylate bands were higher than baseline levels, with values similar to those observed after 14 days of treatment and slightly lower than those obtained with Stela at 28 days (Figure 5).

### 3.2. Energy Dispersive Spectroscopy (EDX)

**Figure 6:**
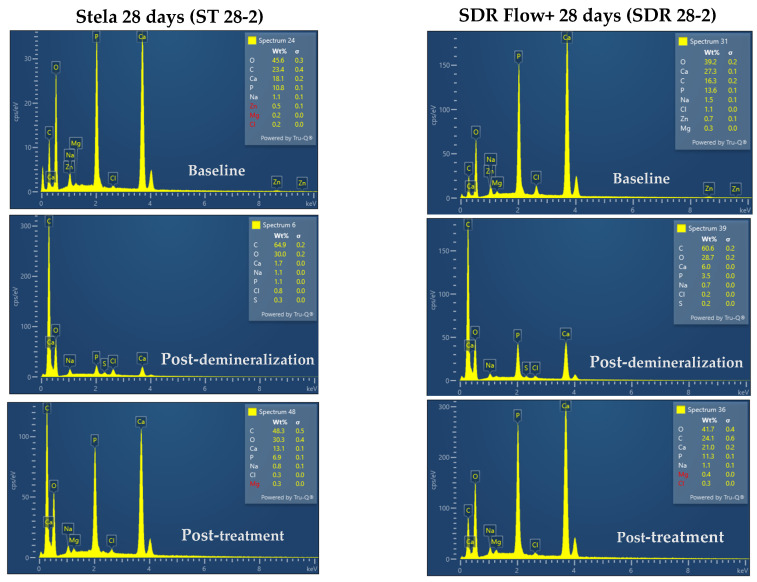
Representative EDX spectra illustrating the elemental analysis methodology employed in the present study. Spectra from a Stela sample (ST 28-2) and SDR Flow+ sample (SDR 28-2) are shown at three time points: baseline, after demineralization with 17% EDTA for 2 h, and after 28 days of material application.

Figure 6 shows representative EDX spectra obtained from one Stela sample and one SDR Flow+ sample at three time points: baseline, after demineralization, and after 28 days of material application. These spectra illustrate the methodology employed for elemental analysis, in which the weight percentages (wt%) of calcium (Ca) and phosphorus (P) were identified and recorded for each sample. This procedure was systematically applied to all samples across all experimental groups and time points, as reported in Table 2 and Table 3.

#### 3.2.1. Comparison Between Demineralization and Treatment According to Exposure Time

The remineralizing ability of Stela and SDR Flow+ was evaluated at four treatment times (1, 7, 14 and 28 days) by analyzing the increase in the mean values of calcium (Ca), phosphorus (P), and the Ca/P ratio between demineralized and treated samples (Table 2).

After 24 h, the Stela-treated group showed increases in Ca, P and Ca/P, with only the increase in the Ca/P ratio being statistically significant (*p* < 0.05). In the SDR Flow+ group, no statistically significant changes were observed in any of the evaluated parameters.

After 7 days of treatment with Stela, the increases in Ca and P were statistically significant (*p* < 0.05), while the Ca/P ratio did not reach statistical significance. In the SDR Flow+ group, both the increases in Ca, P and Ca/P were statistically significant (*p* < 0.05).

At 14 and 28 days, the Stela-treated group showed statistically significant increases in Ca and P (*p* < 0.05), with no statistical significance observed for the Ca/P ratio. In contrast, the SDR Flow+ group exhibited statistically significant increases only in the Ca/P ratio (*p* < 0.05), with no significant changes in the individual Ca and *p* values.

#### 3.2.2. Comparison of Remineralizing Effects Between Stela and SDR Flow+ According to Exposure Time

A Comparison was performed between the remineralizing effect of Stela and SDR Flow+ at four treatment times (1, 7, 14 and 28 days) (Table 3).

At 24 h, no statistically significant differences were observed between the two materials for any of the analyzed variables. Stela showed a greater increase in calcium (Ca) levels and in the Ca/P ratio after treatment, although these differences did not reach statistical significance, while phosphorus (P) values were similar in both groups.

At 7 days, statistically significant differences (*p* < 0.01) were detected between Stela and SDR Flow+ in calcium and phosphorus levels, both in the demineralization phase and after treatment, favoring Stela. However, the post-treatment Ca/P ratio did not show significant differences between the groups.

After 14 days of treatment, SDR Flow+ exhibited more pronounced increases in calcium and phosphorus levels, without reaching statistical significance. However, the Ca/P ratio was significantly higher in the control group (*p* < 0.05).

Finally, at 28 days, both materials showed comparable increases in calcium, phosphorus, and the Ca/P ratio, with no statistically significant differences between the groups.

**Figure 7:**
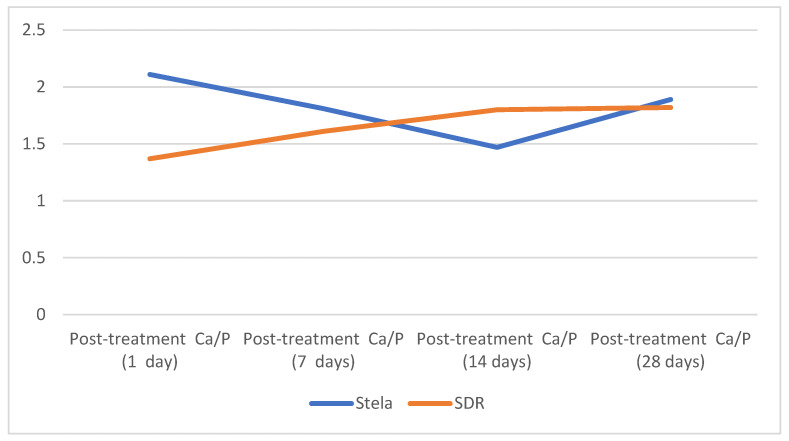
Evolution of post-treatment Ca/P ratio: Stela vs. SDR Flow+.

Figure 7 illustrates the evolution of the Ca/P ratio throughout the treatment period. The *Y*-axis represents the Ca/P expressed as weight percentage (wt%), while the *X*-axis corresponds to the treatment time in days. On the first day, the Stela group showed higher values compared to the control group. However, this ratio decreased over the following days and began to recover from day 14 onwards. In contrast, the SDR Flow+ group started with a lower Ca/P ratio but exhibited a progressive increase throughout the treatment. After 28 days, both groups reached similar Ca/P values.

## 4. Discussion

Minimal intervention dentistry encompasses conservative approaches focused on the early detection of carious lesions and their minimally invasive treatment. This approach aims to prevent caries progression while preserving as much healthy dental tissue as possible [19,20].

In this context, selective caries removal is a less invasive technique that allows the preservation of affected but non-infected dentin, promoting its remineralization [21]. This approach prioritizes remineralization as a key strategy to restore lost dental structure, using bioactive materials capable of inducing favorable responses in dental tissues and promoting the formation of mineralized tissue [22]. Among bioactive materials, remineralizing resins such as SDR Flow+ and Stela have been developed, both incorporating strontium in their composition.

The present study aimed to evaluate the remineralizing potential of a recently introduced composite material (Stela), designed to incorporate bioactive properties. The objective was to assess its behavior in relation to dentin remineralization and to explore whether it may represent a suitable alternative to currently available restorative materials. For this purpose, it was compared with SDR Flow+, a composite resin with a similar composition that has been previously investigated in the literature [23].

Both null hypotheses (H0) were rejected, as the present study suggests that Stela is capable of remineralizing demineralized dentin. Furthermore, significant differences were observed in the dynamics of remineralization and hydroxyapatite formation between Stela and SDR Flow+, depending on the treatment time.

FTIR analysis allowed the evaluation of the remineralizing potential through the identification of different functional groups. The simultaneous presence of peaks associated with phosphates (PO_4_^3−^) and carbonates (CO_3_^2−^) indicates the formation of carbonated hydroxyapatite as an essential component in the remineralization process [17,18].

After 24 h of treatment, Stela appeared to exhibit a greater ability to form carbonated hydroxyapatite than SDR Flow+, whose action appeared more limited. At 7 days, the FTIR spectra of both treatment groups suggest progressive remineralization, without significant differences compared to baseline levels. After 14 days, SDR Flow+ appeared to exhibit greater apatite formation than Stela, although both materials showed considerable increases. At 28 days, Stela maintained its progressive remineralization trend, surpassing initial levels and reaching values similar to SDR Flow+, whereas SDR Flow+ appeared to have reached its maximum efficacy at 14 days, showing no differences in potential apatite formation between 14 days and 28 days.

Overall, both materials appeared to exhibit carbonated apatite after 28 days of treatment, with apparently comparable levels, although the limited sample size warrants caution in this interpretation. These results are consistent with those reported by Ghilotti et al. (2023) [17], who evaluated the remineralization of demineralized dentin using three different glass ionomers. Considering only the 1 mm thickness of the samples used in this study, Stela, as a composite resin incorporating bioactive materials, showed a behavior similar to Ketac Molar (3M ESPE^®^, Seefeld, Germany) in the study by Ghilotti et al.: its remineralization patterns over time were non-linear. In contrast, SDR Flow+ exhibited behavior comparable to Riva Light Cure (SDI^®^ Victoria, Australia), with less pronounced onset of remineralization during the first 24 h compared to Stela, but showing a progressive increase in apatite formation as the treatment increased.

Remineralization is a natural repair process that involves the incorporation of calcium and phosphate to restore the properties of mineralized tissue [2]. Elemental analysis using the FESEM-EDX system allowed quantification of calcium and phosphorus in the samples treated with bioactive resins, enabling the calculation of the Ca/P ratio. This index increases because of dentin remineralization [24].

Stela showed a significant remineralizing effect from the first 24 h, indicating a faster action, although this effect gradually decreased in the following days. In contrast, SDR Flow+ exhibited a notable increase in the Ca/P ratio from day 7, which intensified efficacy in the Ca/P ratio at 28 days, but different dynamics: These preliminary findings suggest that Stela may act more rapidly, whereas SDR Flow+ appears to maintain a more prolonged effect over the medium term, though further studies with larger sample sizes are needed to confirm these dynamics.

Previous studies with resin cements composed of bioactive glass nanoparticles containing strontium and calcium phosphate have shown that higher strontium concentrations promote more efficient release of calcium and strontium ions, enhancing the remineralization process [25]. This occurs because strontium incorporates into the hydroxyapatite crystal lattice by partially substituting calcium ions (Ca^2+^), owing to their chemical similarity. However, the larger ionic radius of Sr^2+^ compared to Ca^2+^ may limit the extent of this substitution within the apatite structure [26]. The resulting strontium-substituted hydroxyapatite exhibits modified physicochemical properties, including altered degradation rates and enhanced bioactivity, which may contribute to the sustained remineralizing effect over time [27]. Additionally, the presence of strontium has been associated with increased fluoride release, thereby improving the chemical stability of the newly formed apatite [10,28]. Consistent with these mechanisms, the biomineralization potential of hydroxyapatite (HAp)-based composites can be confirmed through EDX analysis, where the Ca/P ratio of the mineralized layer approximates the theoretical value of 1.67 for stoichiometric hydroxyapatite [29,30], as was observed in the present study.

Beyond its role in hydroxyapatite crystallization, strontium may also contribute to dentin preservation through the inhibition of matrix metalloproteinases (MMPs). MMPs are a family of zinc- and calcium-dependent endopeptidases that, once activated in an acidic environment such as that generated during caries progression or demineralization, degrade type I collagen—the main component of the dentin organic matrix—thereby compromising the structural integrity of the tissue [5,11]. In this context, Sr^2+^ ions released from strontium-containing composites may interfere with MMP activity through a mechanism analogous to that described for other divalent cations, by competing with the zinc and calcium ions required for their catalytic activity, thus reducing their proteolytic action on the exposed collagen scaffold [31]. Furthermore, strontium has been shown to reduce the expression of MMP-9 and other osteoclast-related metalloproteinases via modulation of the RANKL/OPG pathway, suggesting a broader inhibitory effect on MMP synthesis that extends beyond simple ionic competition [32].

In the context of the present study, this dual mechanism—simultaneous promotion of carbonated hydroxyapatite deposition confirmed by FTIR and EDX, and potential inhibition of collagen-degrading MMPs—is particularly relevant in the clinical scenario of selective caries removal, where the preservation and remineralization of affected dentin are the primary therapeutic goals [21]. The higher strontium-containing filler content of Stela (76 wt%) compared to SDR Flow+ may therefore translate not only into faster initial mineral deposition, as observed in the present results, but also into a more pronounced protective effect on the collagen matrix through MMP inhibition during the early stages of treatment.

Moreover, the combination of strontium and fluoride has a synergistic positive effect on enamel and dentin remineralization, promoting crystallization and reducing hydroxyapatite dissolution in acidic environments. Strontium may also stimulate cellular differentiation of dental pulp cells toward a dentin-like matrix, providing additional benefits for regenerative dental applications [33,34].

Relating these findings to the results of the present study, Stela, which has a higher filler content including strontium, appears to induce faster remineralization. However, both Stela and SDR Flow+ maintain a prolonged remineralizing effect.

Finally, no significant differences were found in the Ca/P ratio between baseline and post-treatment measurements for either material at any of the evaluated time points. The results are consistent with those reported in a previous study performed by our research group for glass ionomers, where at thicknesses of 1 and 2 mm, these bioactive materials were able to restore values to levels prior to demineralization.

These preliminary results suggest that Stela and SDR Flow+ may facilitate the incorporation of calcium and phosphorus into demineralized dentin over time, potentially providing remineralizing properties similar to those of glass ionomer. However, their aesthetic and mechanical advantages as composite resins would need to be confirmed through additional mechanical and biological testing.

One factor that may also contribute to the faster initial remineralizing effect of Stela is that its application involves only a primer, whereas SDR Flow+ requires a universal adhesive system. It is well established that adhesive layers—particularly those derived from universal adhesive systems—can act as diffusion barriers at the resin-dentin interface, limiting the penetration of ions and fluids into the underlying demineralized tissue [35,36]. However, the findings of this study suggest that, although this barrier may slow ion diffusion, it does not appear to prevent remineralization under the conditions evaluated, as evidenced by the progressive increase in the Ca/P ratio and apatite formation observed in the SDR Flow+ group over time.

This study has several limitations that should be considered when interpreting the results. Regarding the demineralization model, 17% EDTA was selected as a widely used and well-established agent for inducing controlled dentin demineralization in in vitro studies, due to its high reproducibility and experimental standardization [36]. However, it should be acknowledged that EDTA acts primarily as a chelating agent that removes calcium ions from the hydroxyapatite lattice and, therefore, does not fully replicate the dynamic acid-mediated demineralization and remineralization cycles that characterize the natural caries process in the oral environment [37,38]. More clinically representative models, such as pH-cycling protocols or bacterial biofilm models, would provide a closer approximation to real caries conditions and are recommended for future studies.

Regarding the analytical methods, the FTIR analysis performed in this study was primarily qualitative in nature, based on the visual identification and interpretation of spectral peaks. While this approach is consistent with the methodology reported in comparable studies [17], it does not allow for accurate quantification of mineral content. Future studies should consider incorporating quantitative spectral analysis techniques, such as peak deconvolution or the calculation of mineral-to-matrix ratios, to provide more robust and comparable data. Similarly, only one FTIR measurement per sample was performed, which limits the evaluation of intra-sample variability.

The EDX analysis also showed considerable variability, reflected in large standard deviations, which is likely attributable to the small sample size (*n* = 3 per group). This limitation increases the likelihood of both type I and type II errors, thereby reducing the statistical robustness of the comparisons. In addition, the presence of zero values in many demineralized samples may have influenced the statistical analyses, potentially underestimating true differences between groups.

Furthermore, although the EDX measurement area was standardized across all samples by consistently selecting the dentin surface opposite to the composite application site, dentin surfaces may present inherent structural heterogeneity at the microscopic level, which could influence the EDX reading obtained.

Regarding the storage medium, PBS was used to maintain the samples during the observation period. While PBS provides a standardized and reproducible environment widely employed in comparable in vitro studies [16], it does not fully replicate the complex ionic composition of saliva or simulated body fluid (SBF), which contains additional ions and proteins that may influence the remineralization process in vivo. Future studies should consider the use of artificial saliva or SBF to better approximate the intraoral environment.

Given these constraints, the present study should be considered exploratory in nature. Future studies with larger sample sizes, quantitative spectral analysis, and more biologically relevant demineralization models are warranted to confirm and expand upon these findings.

## 5. Conclusions

FTIR and FESEM-EDX analyses indicate that both materials promoted remineralization of demineralized dentin, as evidenced by carbonated hydroxyapatite formation and increases in calcium and phosphate levels over time. Stela showed an early remineralizing effect, with a significant increase in the Ca/P ratio after 24 h (2.11 ± 0.41; *p* < 0.05), whereas SDR Flow+ exhibited a more progressive response, reaching peak Ca/P values at 14 days (1.81 ± 0.18; *p* < 0.01) and remaining stable thereafter. Despite these differences in remineralization dynamics, both materials achieved apparently comparable results at the end of the study (*p* > 0.05). However, given the limitations of the in vitro model and sample size, these findings should be interpreted with caution, and future studies with larger sample sizes, quantitative spectral analysis, and biological validation are needed to confirm the remineralizing potential of the materials under clinically relevant conditions.

## Figures and Tables

**Table 1:** Composition and Manufacturer of the Composites.

Material	Composition	Manufacturer	Batch Number
Stela	Matrix: Methacrylates (23 wt%; 44 vol%): urethane dimethacrylate (UDMA) and glycerol dimethacrylate (GDM). Fillers: 76 wt% (55 vol%): fluoroaluminosilicate glass, ytterbium trifluoride, silicon dioxide, calcium aluminate, strontium fillers. Initiators, Stabilizer, Pigments: <1 wt% each	SDI	1241102
SDR Flow+	Resin Matrix: Urethane dimethacrylate, modified EBPADMA, TEGDMA. Inorganic Fillers: 20 nm–10 µm; 47.3 vol%: barium-aluminum-fluoruro-borosilicate crystals, strontium fluoro-silicate crystals. Additives: photoinitiator, photo accelerator, BHT, UV stabilizer, titanium dioxide, iron oxide pigments, fluorescent agent.	Dentsply Sirona	00153481

**Table 2:** Demineralization vs. treatment according to exposure time.

Day	Material	Variable	Post-Demineralization	Post-Treatment	*p* Value
1	Stela	(Ca)	1.30 ± 2.25	13.20 ± 15.31	0.12
		(P)	0.97 ± 1.67	6.50 ± 6.97	0.11
		(Ca/P)	0.45 ± 0.78	2.11 ± 0.41	0.03 *
	SDR	(Ca)	4.67 ± 8.08	9.60 ± 8.29	0.16
		(P)	2.87 ± 4.97	5.60 ± 3.68	0.17
		(Ca/P)	1.54 ± 0.94	1.38 ± 0.62	0.11
7	Stela	(Ca)	1.60 ± 0.30	7.73 ± 1.27	0.01 *
		(P)	1.00 ± 0.20	4.30 ± 0.46	0.01 *
		(Ca/P)	0.43 ± 0.75	1.81 ± 0.36	0.16
	SDR	(Ca)	0.00 ± 0.00	0.30 ± 0.10	0.01 *
		(P)	0.00 ± 0.00	0.20 ± 0.10	0.03 *
		(Ca/P)	0.00 ± 0.00	1.61 ± 0.35	<0.05 *
14	Stela	(Ca)	0.63 ± 0.46	5.57 ± 1.80	0.04 *
		(P)	0.47 ± 0.32	3.77 ± 1.26	0.04 *
		(Ca/P)	0.50 ± 0.87	1.48 ± 0.09	0.20
	SDR	(Ca)	1.53 ± 1.46	15.03 ± 8.02	0.05
		(P)	1.10 ± 0.85	8.06 ± 3.78	0.05
		(Ca/P)	0.00 ± 0.00	1.81 ± 0.18	<0.01 **
28	Stela	(Ca)	4.87 ± 3.15	13.36 ± 3.50	0.02 *
		(P)	8.50 ± 2.02	2.87 ± 1.86	0.02 *
		(Ca/P)	0.56 ± 0.96	1.89 ± 0.15	0.08
	SDR	(Ca)	4.07 ± 3.52	9.60 ± 6.03	0.23
		(P)	2.23 ± 1.94	5.03 ± 3.65	0.22
		(Ca/P)	0.00 ± 0.14	1.82 ± 0.14	<0.001 **

* *p* < 0.05; ** *p* < 0.01.

**Table 3:** Comparative analysis of remineralizing effects between Stela and SDR Flow+ according to exposure time.

Variable	Day	Condition	Stela	SDR	*p* Value
(Ca)	1	Post-demineralization	8.50 ± 10.22	13.90 ± 10.65	0.33
		Post-treatment	13.20 ± 15.31	9.60 ± 8.29	0.36
	7	Post-demineralization	0.20 ± 0.00	0.20 ± 0.10	1.00
		Post-treatment	7.73 ± 1.27	0.30 ± 0.1	<0.01 **
	14	Post-demineralization	10.50 ± 6.70	8.63 ± 6.65	0.59
		Post-treatment	5.57 ± 1.80	15.03 ± 8.02	0.05
	28	Post-demineralization	4.33 ± 4.14	5.66 ± 5.61	0.61
		Post-treatment	13.36 ± 3.50	9.60 ± 6.03	0.3
(P)	1	Post-demineralization	5.30 ± 6.18	6.10 ± 4.75	0.77
		Post-treatment	6.50 ± 6.97	5.60 ± 3.68	0.43
	7	Post-demineralization	0.10 ± 0.00	0.10 ± 0.00	1.00
		Post-treatment	4.30 ± 0.46	0.20 ± 0.1	<0.01 **
	14	Post-demineralization	4.77 ± 3.11	4.53 ± 3.55	0.90
		Post-treatment	3.77 ± 1.26	8.06 ± 3.78	0.06
	28	Post-demineralization	1.90 ± 1.80	2.70 ± 2.75	0.52
		Post-treatment	2.87 ± 1.86	5.03 ± 3.65	0.28
(Ca/P)	1	Post-demineralization	1.59 ± 0.19	2.18 ± 0.17	<0.01 **
		Post-treatment	2.11 ± 0.41	1.38 ± 0.62	0.08
	7	Post-demineralization	2.00 ± 0.00	2.00 ± 0.00	1.00
		Post-treatment	1.81 ± 0.36	1.61 ± 0.35	0.25
	14	Post-demineralization	2.18 ± 0.10	1.88 ± 0.09	<0.01 **
		Post-treatment	1.48 ± 0.09	1.81 ± 0.18	0.02 *
	28	Post-demineralization	2.23 ± 0.11	2.05 ± 0.08	0.01 *
		Post-treatment	1.89 ± 0.15	1.82 ± 0.14	0.29

* *p* < 0.05; ** *p* < 0.01.

## Data Availability

The original contributions presented in this study are included in the article. Further inquiries can be directed to the corresponding author.
